# 137. Penicillin (PCN) Gradient Diffusion Overcalls Resistance and Promotes Unnecessary Vancomycin (VAN) Use in *Enterococcus faecalis* Bloodstream Infections (BSI)

**DOI:** 10.1093/ofid/ofad500.210

**Published:** 2023-11-27

**Authors:** Lindsay E Donohue, Heather L Cox, Jenni Thomas, Tiffany S Kidd, Hiba N Mohammed, Amy Mathers

**Affiliations:** University of Virginia Health, Keswick, Virginia; University of Virginia Health, Keswick, Virginia; University of Virginia Health, Keswick, Virginia; University of Virginia Health, Keswick, Virginia; University of Virginia Health system, Charlottesville, Virginia; University of Virginia, Charlottesville, VA

## Abstract

**Background:**

Reports of a PCN-resistant (R), ampicillin-susceptible (S) *Enterococcus faecalis* (PRASEF) phenotype associated with elevated piperacillin and imipenem minimum inhibitory concentrations (MICs) may drive hesitation to use these agents when a PCN MIC is unknown and/or result in the addition of a glycopeptide. However, PCN MICs by broth microdilution are not readily available in many clinical laboratories and some commercial methods may overcall PRASEF. We sought to assess the rate of overcalling PCN MICs by gradient diffusion compared to broth microdilution and the impact of PCN test requests and results on vancomycin (VAN) prescribing attributed to possible PRASEF BSI at a single academic medical center.

**Methods:**

Retained clinical *E. faecalis* blood isolates with Etest® (BioMérieux, Inc.) PCN MICs collected from 2019 – 2022 were tested via automated broth microdilution (aBMD) plates (Sensititre™ Thermo Fisher Scientific, Inc.). MICs were interpreted according to CLSI breakpoints and compared for essential agreement (EA) and categorical agreement (CA) of Etest to aBMD. A retrospective chart review of all PCN-S or PRASEF BSI from 1/2021 – 12/2022 identified via antibiogram report generation (Theradoc® Premier, Inc.) plus all patients with a retained isolate was conducted to measure VAN days of therapy (DOT) attributed to concern for PRASEF.

**Results:**

Of the 5 PCN-S and 19 PCN-R isolates via Etest MIC for which aBMD was performed, all tested PCN-S with an MIC50/90 of 4/8 mcg/mL. EA was 33% and CA was 21%, with a 79% major error rate (called resistant when susceptible). A total of 124 patient charts were reviewed (n=25 Etest PCN-R, n=99 Etest PCN-S). Total attributable VAN DOT was 102 across 19 patients (n=3 Etest PCN-R, n=16 Etest PCN-S), with a mean VAN DOT of 17 in PCN-R and 3 in PCN-S isolates.

**Conclusion:**

False resistant PCN Etest MICs, as determined by aBMD comparison and consistent with published reports, fed concern regarding potential PRASEF emergence at our institution and led to unnecessary concomitant VAN in 15% of patients. Caution is advised when interpreting PCN susceptibility via this method. Inclusion of *E. faecalis* in the FDA indication for *in vitro* diagnostic use of benzylPCN Etest should be reconsidered.

**Disclosures:**

**Amy Mathers, MD, D(ABMM)**, Merck: Advisor/Consultant

